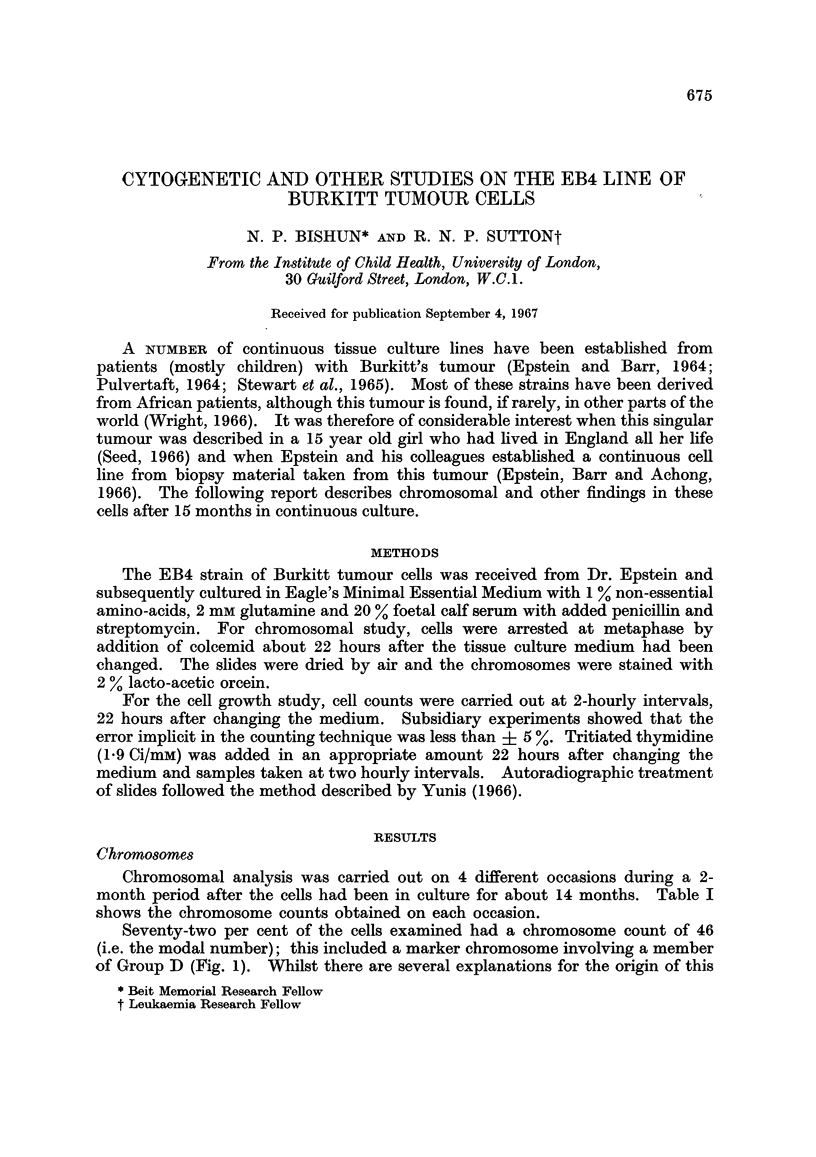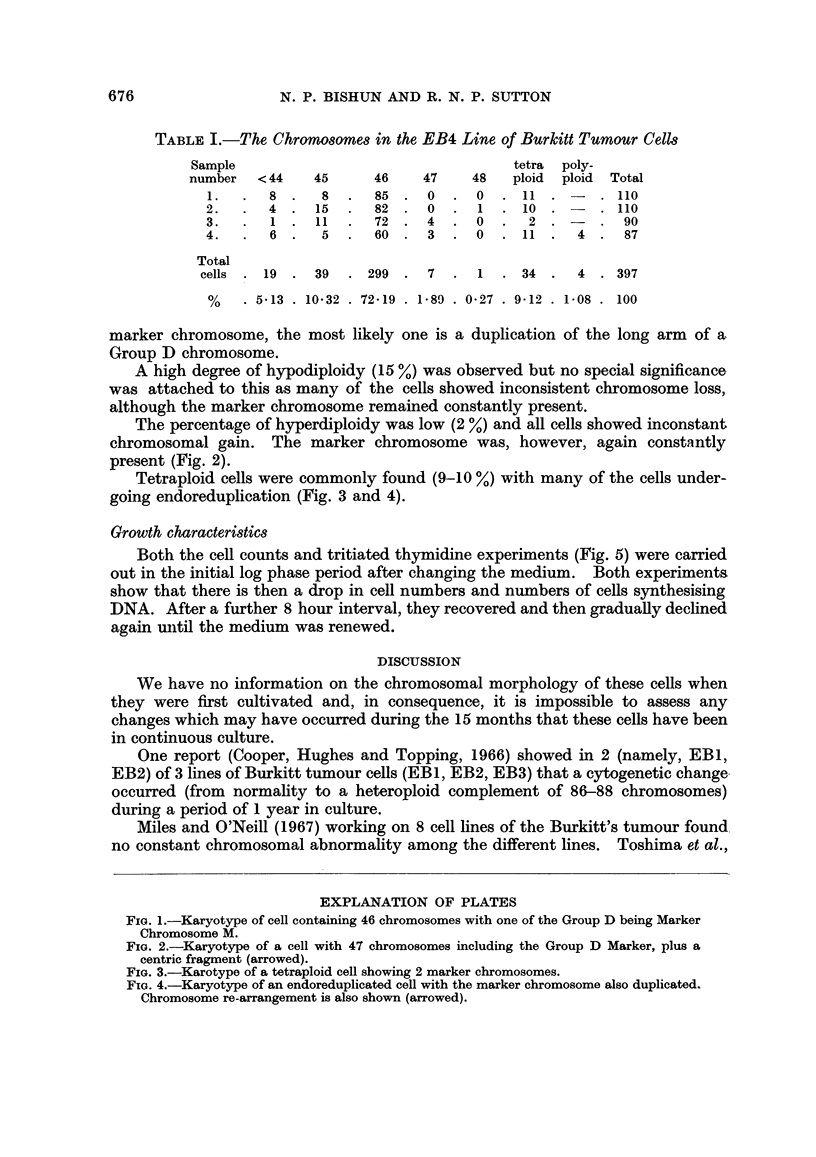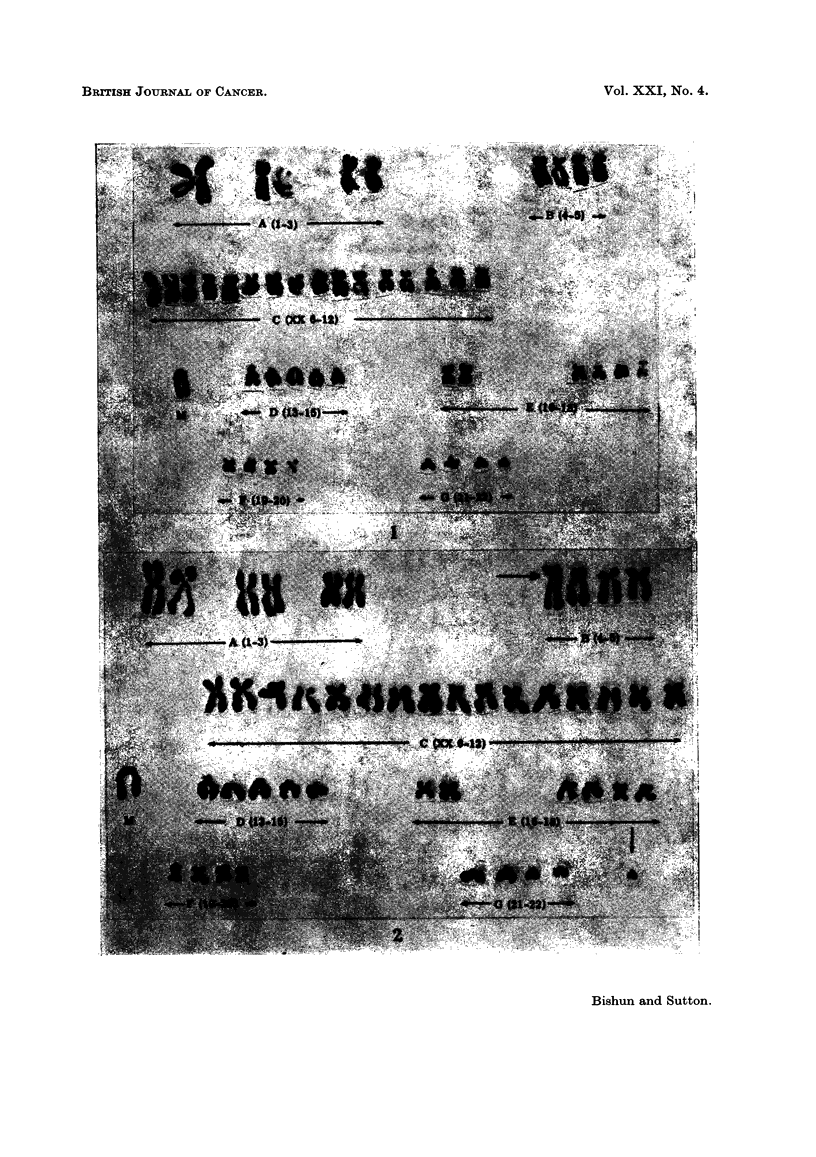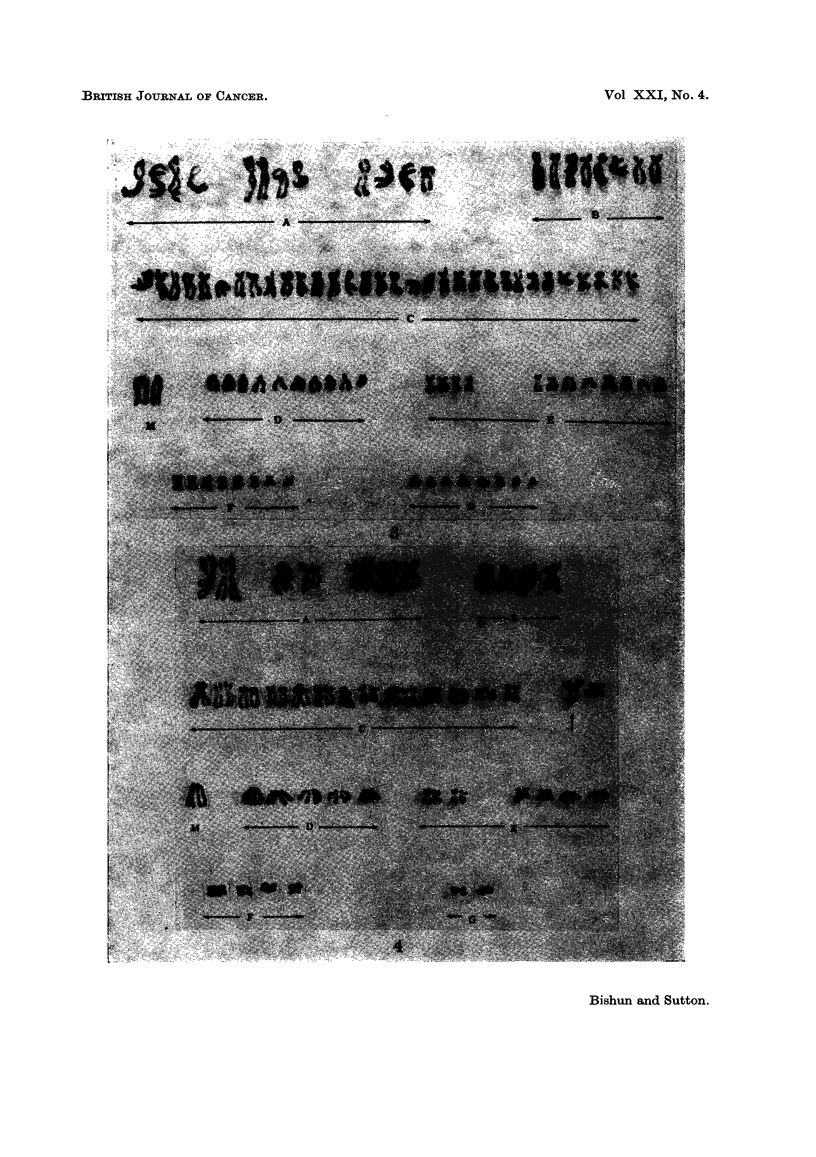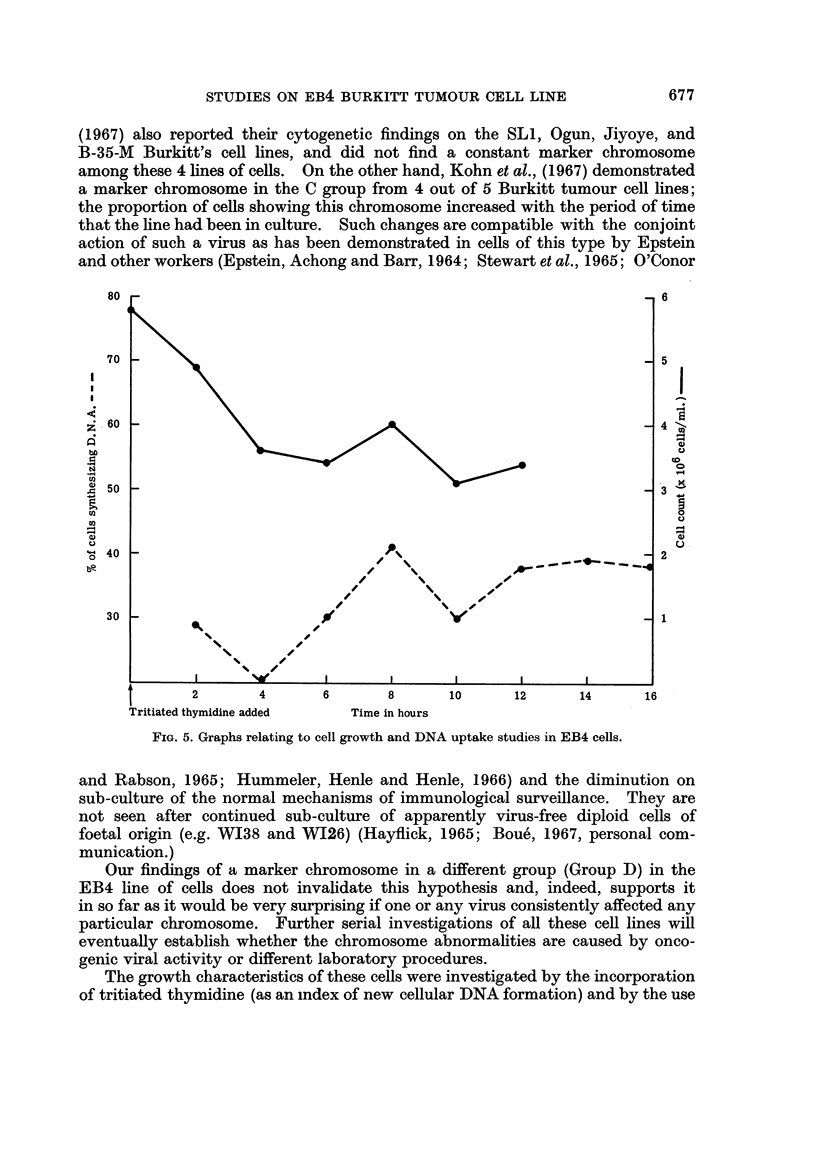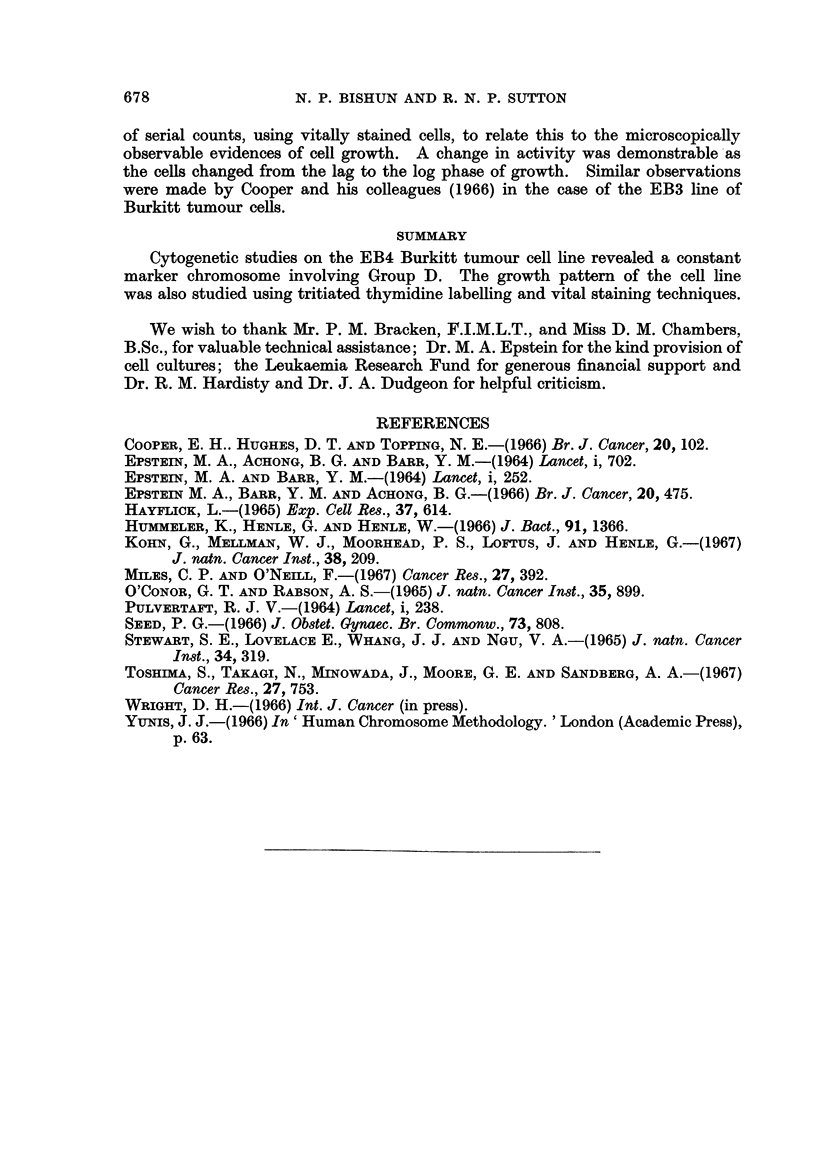# Cytogenetic and other studies on the EB4 line of Burkitt tumour cells.

**DOI:** 10.1038/bjc.1967.79

**Published:** 1967-12

**Authors:** N. P. Bishun, R. N. Sutton

## Abstract

**Images:**


### CYTOGENETIC AND OTHER STUDIES ON THE EB4 LINE OF BURKITT TUMOUR CELLS

N. P. BISHUN* AND R. N. P. SUTTON†

*From the Institute of Child Health, University of London, 30 Guilford Street, London, W.C.1.*

Received for publication September 4, 1967

A NUMBER of continuous tissue culture lines have been established from patients (mostly children) with Burkitt's tumour (Epstein and Barr, 1964; Pulvertaft, 1964; Stewart *et al.*, 1965). Most of these strains have been derived from African patients, although this tumour is found, if rarely, in other parts of the world (Wright, 1966). It was therefore of considerable interest when this singular tumour was described in a 15 year old girl who had lived in England all her life (Seed, 1966) and when Epstein and his colleagues established a continuous cell line from biopsy material taken from this tumour (Epstein, Barr and Achong, 1966). The following report describes chromosomal and other findings in these cells after 15 months in continuous culture.

### METHODS

The EB4 strain of Burkitt tumour cells was received from Dr. Epstein and subsequently cultured in Eagle's Minimal Essential Medium with 1 % non-essential amino-acids, 2 mM glutamine and 20 % foetal calf serum with added penicillin and streptomycin. For chromosomal study, cells were arrested at metaphase by addition of colcemid about 22 hours after the tissue culture medium had been changed. The slides were dried by air and the chromosomes were stained with 2 % lacto-acetic orcein.

For the cell growth study, cell counts were carried out at 2-hourly intervals, 22 hours after changing the medium. Subsidiary experiments showed that the error implicit in the counting technique was less than ± 5 %. Tritiated thymidine (1·9 Ci/mM) was added in an appropriate amount 22 hours after changing the medium and samples taken at two hourly intervals. Autoradiographic treatment of slides followed the method described by Yunis (1966).

### RESULTS

### *Chromosomes*

Chromosomal analysis was carried out on 4 different occasions during a 2-month period after the cells had been in culture for about 14 months. Table I shows the chromosome counts obtained on each occasion.

Seventy-two per cent of the cells examined had a chromosome count of 46 (i.e. the modal number); this included a marker chromosome involving a member of Group D (Fig. 1). Whilst there are several explanations for the origin of this

* Beit Memorial Research Fellow
† Leukaemia Research Fellow

TABLE I.—*The Chromosomes in the EB4 Line of Burkitt Tumour Cells*

| Sample number | <44 | 45 | 46 | 47 | 48 | tetra ploid | poly-ploid | Total |
|---|---|---|---|---|---|---|---|---|
| 1. | 8 | 8 | 85 | 0 | 0 | 11 | — | 110 |
| 2. | 4 | 15 | 82 | 0 | 1 | 10 | — | 110 |
| 3. | 1 | 11 | 72 | 4 | 0 | 2 | — | 90 |
| 4. | 6 | 5 | 60 | 3 | 0 | 11 | 4 | 87 |
| Total cells | 19 | 39 | 299 | 7 | 1 | 34 | 4 | 397 |
| % | 5·13 | 10·32 | 72·19 | 1·89 | 0·27 | 9·12 | 1·08 | 100 |

marker chromosome, the most likely one is a duplication of the long arm of a Group D chromosome.

A high degree of hypodiploidy (15 %) was observed but no special significance was attached to this as many of the cells showed inconsistent chromosome loss, although the marker chromosome remained constantly present.

The percentage of hyperdiploidy was low (2 %) and all cells showed inconstant chromosomal gain. The marker chromosome was, however, again constantly present (Fig. 2).

Tetraploid cells were commonly found (9–10 %) with many of the cells undergoing endoreduplication (Fig. 3 and 4).

*Growth characteristics*

Both the cell counts and tritiated thymidine experiments (Fig. 5) were carried out in the initial log phase period after changing the medium. Both experiments show that there is then a drop in cell numbers and numbers of cells synthesising DNA. After a further 8 hour interval, they recovered and then gradually declined again until the medium was renewed.

### DISCUSSION

We have no information on the chromosomal morphology of these cells when they were first cultivated and, in consequence, it is impossible to assess any changes which may have occurred during the 15 months that these cells have been in continuous culture.

One report (Cooper, Hughes and Topping, 1966) showed in 2 (namely, EB1, EB2) of 3 lines of Burkitt tumour cells (EB1, EB2, EB3) that a cytogenetic change occurred (from normality to a heteroploid complement of 86–88 chromosomes) during a period of 1 year in culture.

Miles and O'Neill (1967) working on 8 cell lines of the Burkitt's tumour found no constant chromosomal abnormality among the different lines. Toshima *et al.*,

---

### EXPLANATION OF PLATES

FIG. 1.—Karyotype of cell containing 46 chromosomes with one of the Group D being Marker Chromosome M.

FIG. 2.—Karyotype of a cell with 47 chromosomes including the Group D Marker, plus a centric fragment (arrowed).

FIG. 3.—Karotype of a tetraploid cell showing 2 marker chromosomes.

FIG. 4.—Karyotype of an endoreduplicated cell with the marker chromosome also duplicated. Chromosome re-arrangement is also shown (arrowed).

1

2

Bishun and Sutton.

3

4

Bishun and Sutton.

(1967) also reported their cytogenetic findings on the SL1, Ogun, Jiyoye, and B-35-M Burkitt's cell lines, and did not find a constant marker chromosome among these 4 lines of cells. On the other hand, Kohn *et al.*, (1967) demonstrated a marker chromosome in the C group from 4 out of 5 Burkitt tumour cell lines; the proportion of cells showing this chromosome increased with the period of time that the line had been in culture. Such changes are compatible with the conjoint action of such a virus as has been demonstrated in cells of this type by Epstein and other workers (Epstein, Achong and Barr, 1964; Stewart *et al.*, 1965; O'Conor

FIG. 5. Graphs relating to cell growth and DNA uptake studies in EB4 cells.

and Rabson, 1965; Hummeler, Henle and Henle, 1966) and the diminution on sub-culture of the normal mechanisms of immunological surveillance. They are not seen after continued sub-culture of apparently virus-free diploid cells of foetal origin (e.g. WI38 and WI26) (Hayflick, 1965; Boué, 1967, personal communication.)

Our findings of a marker chromosome in a different group (Group D) in the EB4 line of cells does not invalidate this hypothesis and, indeed, supports it in so far as it would be very surprising if one or any virus consistently affected any particular chromosome. Further serial investigations of all these cell lines will eventually establish whether the chromosome abnormalities are caused by oncogenic viral activity or different laboratory procedures.

The growth characteristics of these cells were investigated by the incorporation of tritiated thymidine (as an index of new cellular DNA formation) and by the use of serial counts, using vitally stained cells, to relate this to the microscopically observable evidences of cell growth. A change in activity was demonstrable as the cells changed from the lag to the log phase of growth. Similar observations were made by Cooper and his colleagues (1966) in the case of the EB3 line of Burkitt tumour cells.

### SUMMARY

Cytogenetic studies on the EB4 Burkitt tumour cell line revealed a constant marker chromosome involving Group D. The growth pattern of the cell line was also studied using tritiated thymidine labelling and vital staining techniques.

We wish to thank Mr. P. M. Bracken, F.I.M.L.T., and Miss D. M. Chambers, B.Sc., for valuable technical assistance; Dr. M. A. Epstein for the kind provision of cell cultures; the Leukaemia Research Fund for generous financial support and Dr. R. M. Hardisty and Dr. J. A. Dudgeon for helpful criticism.

### REFERENCES

COOPER, E. H.. HUGHES, D. T. AND TOPPING, N. E.—(1966) *Br. J. Cancer*, **20,** 102.
EPSTEIN, M. A., ACHONG, B. G. AND BARR, Y. M.—(1964) *Lancet*, i, 702.
EPSTEIN, M. A. AND BARR, Y. M.—(1964) *Lancet*, i, 252.
EPSTEIN M. A., BARR, Y. M. AND ACHONG, B. G.—(1966) *Br. J. Cancer*, **20,** 475.
HAYFLICK, L.—(1965) *Exp. Cell Res.*, **37,** 614.
HUMMELER, K., HENLE, G. AND HENLE, W.—(1966) *J. Bact.*, **91,** 1366.
KOHN, G., MELLMAN, W. J., MOORHEAD, P. S., LOFTUS, J. AND HENLE, G.—(1967) *J. natn. Cancer Inst.*, **38,** 209.
MILES, C. P. AND O'NEILL, F.—(1967) *Cancer Res.*, **27,** 392.
O'CONOR, G. T. AND RABSON, A. S.—(1965) *J. natn. Cancer Inst.*, **35,** 899.
PULVERTAFT, R. J. V.—(1964) *Lancet*, i, 238.
SEED, P. G.—(1966) *J. Obstet. Gynaec. Br. Commonw.*, **73,** 808.
STEWART, S. E., LOVELACE E., WHANG, J. J. AND NGU, V. A.—(1965) *J. natn. Cancer Inst.*, **34,** 319.
TOSHIMA, S., TAKAGI, N., MINOWADA, J., MOORE, G. E. AND SANDBERG, A. A.—(1967) *Cancer Res.*, **27,** 753.
WRIGHT, D. H.—(1966) *Int. J. Cancer* (in press).
YUNIS, J. J.—(1966) *In* ' Human Chromosome Methodology. ' London (Academic Press), p. 63.